# Automatic Optical Measurement and Control of Benzene and Benzenoids in Natural Gas Pipelines

**DOI:** 10.3390/s21227575

**Published:** 2021-11-15

**Authors:** Rossana Galassi, Christian Contini, Matteo Pucci, Ennio Gambi

**Affiliations:** 1 School of Science and Technology, Chemistry Division, University of Camerino, 62032 Camerino, Italy; rossana.galassi@unicam.it; 2 Automa Srl, 60131 Ancona, Italy; christian.contini@byautoma.com (C.C.); matteo.pucci@byautoma.com (M.P.); 3 Department of Information Engineering, Polytechnic University of Marche, 60131 Ancona, Italy

**Keywords:** natural gas measurement, pipeline monitoring, benzene, benzenoids

## Abstract

The presence of benzene and similar aromatic compounds in civil environments is due to anthropic actions but also to natural sources. Natural gas consists of a gas mixture where benzene and related compounds are usually presents. Thus, the detection of these compounds in natural gas pipelines is of the utmost importance as well as the control of the concentration level, which must remain below the limits consented by law. In this regard, it is of striking interest to engineer devices able to detect these compounds by automatic and continuous remote control. Here, we discuss the application of an optical device designed for the measurement of sulfured odorizing agents in natural gas pipelines aiming at the detection and the measurement of benzene, toluene, and xylenes (BTX) in the same contexts. The instrument consists of a customized UV spectrophotometer connected to an automatic control system able to provide in-field detections of BTX through a continuous and remote check of the gaseous mixture. Relatively to benzene, the instrument is characterized by values of LOD (level of detection) and LOQ (level of quantification) equal to 0.55 and 1.84 mg/Sm${}^{3}$, respectively. Similar limits are found for toluene and xylenes (LOD of 0.81, 1.05, 1.41, and 1.00 mg/Sm${}^{3}$ for toluene, meta-, ortho-, and para-xylene, respectively).

## 1. Introduction

Natural gas pipelines are spread worldwide given that natural gas is a common fuel for civil purposes. Since natural gas is such a widespread energy source, the safety of the pipelines and of their sorting/decompression booths is critical both for the potential explosive hazard and for the atmospheric pollution due to the occurrence of leakages [1]. The composition of natural gas is extremely variable and aside from the bulk presence of aliphatic hydrocarbons, many other components are present from ppm to ppb concentrations; among them, sulfur derivatives, oxygen, nitrogen, carbon dioxide, carbon monoxide, and the undesirable aromatic hydrocarbons such as benzene, toluene, and xylenes (BTX) are worth mentioning [2,3]. The detection of benzene and its related compounds is a big issue in the field of environmental control and of safety in public and private areas or in workplace locations [4]. In biomass conversion plants, the presence of this kind of dangerous compound in the obtained gaseous fuel mixture may also stiffly rise up and significant amounts may be dispersed in the atmosphere [5]. Therefore, obtaining a continuous, reliable, remote control for the presence of these compounds is a need that cannot be postponed. Some studies have been devoted to the search of methods of analysis responding to the demand of sensors that are portable, low cost, and applicable to different circumstances [6]. Most of the apparatus are based on electrochemical [7] and metal oxide sensors, micro-gas chromatographs (μGC) [8], or electronic noses [9,10] with variable limit of detection (generally quite low) but with significant problems in calibration and selectivity. Detection methods for the measurement of benzene and benzenoids have been based on AAS (atomic absorption spectroscopy) [11] or by applying infrared spectroscopy [12]. Some IR studies have been recently addressed to the detection of sub-ppm concentrations of benzene, with experimental detection limits of 0.26 and 0.41 ppm for benzene and toluene (using a 100 m path length for these two gases at atmospheric pressure) [13]. Moreover, some colorimetric assays have been developed by using sensitive materials [14] and a simple fluorescent sensor [15] has also been developed utilizing a material based on dibenzoylmethanatoboron difluoride (DBMBF2), a fluorophore which is capable of forming exciplexes with benzene, toluene, and xylenes (BTX). Upon calibration, the fluorescent sensor provides simultaneous quantification of benzene, toluene, and p-xylene in three-component mixtures [15]. As UV–visible spectroscopy is concerned, this technique is already applied to the detection of benzene as the detectors for MicroGC spectrometers are based on this technique. The UV–visible detector directly measures the absorbance values in volts using an integrated circuit with a log-ratio amplifier. A portable UV–visible detector prototype was tested in tanks with nitrogen and benzene or toluene-$N_{2}$ (1.5 to 50 ppm), and good linearity ($R^{2}$ = 0.99) with a limit of detection of 196 ppb was obtained [16]. The allowed limit for benzene in natural gas pipelines in Europe and the UK is dictated by the Commission Regulation (EU) 2015/1494 imposing a maximum concentration of benzene in natural gas equal to 0.1% v/v [17]. So far, in the literature, there are no systems based on UV–visible spectroscopy for the on-line and automatic determination of the aromatic hydrocarbons content in natural gas. In general, in the market, it seems to be a severe lack of systems that can operate directly in field. In this work, we propose an innovative UV–visible spectroscopy-based instrument aimed at the detection and measurement of the content of benzene, toluene, and total xylenes in the pipeline with a remote control approach. This solution requires a low energy cost and a low frequency of maintenance carried out by specialized personnel for the renewal of consumables.

The paper is organized as follows. In Section 2, some detail about instrumentation and method of analysis are provided, while in Section 3, results are presented and discussed. Finally, Section 4 concludes the paper.

## 2. Experimental Section

### 2.1. Instrumentation

**Figure 1:**
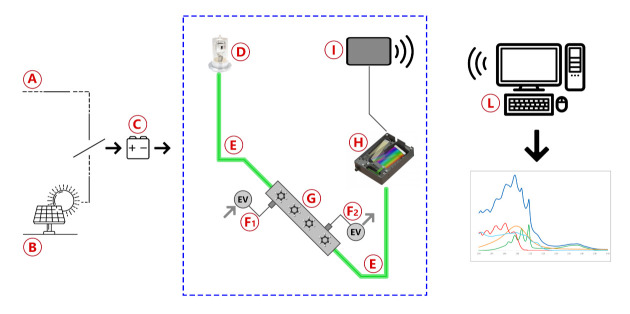
Blocks diagram of the instrument used for the measure of benzene and benzenoids in natural gas pipelines.

The Spectra instrument is schematically represented in Figure 1 and consists of a prototype of a UV—vis spectrophotometer adapted for the in-field sampling and analysis of pipeline gas. The main blocks are the UV deuterium lamp, a stainless-steel flow cell with built-in focusing lenses, a commercial dispersive block, and two optical fibers with a 600 μm quartz core.

As depicted in Figure 1, the system is powered by either mains (A) or solar cells (B) and uses a common 14V battery (C) as a backup power source in case of brief power shortages and as a buffer to balance high and low solar cell insulation conditions. The light produced by a deuterium lamp (D) is delivered through the sample into the detector by two optical fibers (E). Gas flow is controlled by two solenoid valves, with one acting as the gas inlet (F1) and the other one acting as the gas outlet (F2), in order to fill the flow cell (G), alternately, with reference gas and with sample gas. Light transmitted through the gas is analyzed by a spectrometer (H) and the data collected is sent by a wireless communication device (I) to a remote data processing unit (L) to obtain spectra and concentration results. Although the spectrometer used can analyze light from 180 to 450 nm, the wavelength range of analysis is usually reduced to 200–280 nm to obtain the best possible resolution in the region of interest. The gas inlet consists of a particulate filter, valves to regulate the pressure, and a pump to regulate the gas flow inside the cell. The measurements were made by comparing the acquisitions of pipeline gas with those obtained from standard canisters at known concentrations (normed according to ISO 6143) and the calibration line previously built. After the installation of Spectra on a pipeline, a validation of the results was made by a comparative analysis, regarding the measurement performed by Spectra and those of two independent laboratories (named Lab A and Lab B in Table 1). In the labs, different methods of analysis were used: the GC after solvent extraction of an adsorbed cartridge and a colorimetric assay. The data referred to the analyses of benzene in nitrogen spilled from a 52 mg/m${}^{3}$ standard canister are reported in Table 1. The goodness of the data supports the validity of the proposed approach. Occasionally, validation of the results was performed by comparison with certified portable gas-chromatographic systems in situ, as described in a previous work for the validation of odorant concentration measurements [18]. The whole measuring system is currently patent pending.

The analyses of benzene and benzenoids were led by keeping the gas sample at 2 bar pressure and at room temperature. Each analysis begins with the automatized cleaning of the gas cell, followed by the acquisition of the light transmitted through a reference gas introduced after the cleaning, allowing to take into account the background of the measurement (blank); afterward the analytical sample is introduced by the valves system and the light transmitted through it is acquired (sample). The method of analysis is based on the well-known Lambert–Beer’s Law, where the absorbance of a gas sample, $A_{sample}$, can be calculated as follows:

(1) $$A_{sample}=-log\frac{I_{sample}}{I_{0}}$$

$I_{sample}$ is the intensity of light transmitted through the sample and $I_{0}$ is the intensity of the incident light. To overcome the technical issues of the $I_{0}$ measurement, an indirect method of analysis with the use of a reference gas (blank) was chosen. Consequently, by referring the absorbance of the natural gas mixture to a blank, the experimental absorbance, $A_{experimental}$, may be derived, according to Equation 1, from the following analytical relationships:

(2) $$A_{sample}-A_{blank}=A_{experimental}=-log\frac{I_{sample}}{I_{0}}-\left(-log\frac{I_{blank}}{I_{0}}\right)=-log\frac{I_{sample}}{I_{blank}}$$

The reference gas and alkanes are non-absorbing in the range of interest (200–280 nm); therefore, $A_{experimental}$ corresponds to $A_{sample}$ and it can be calculated by the ratio of the light transmitted through the gas mixture and the light transmitted through the reference gas. Conversely, if the reference gas shows absorptions in the observed range and it is not a component of the mixture, the attained spectrum might display negative bands, according to Equation (2). Atmospheric air was chosen as the reference gas because it does not show significant absorptions at wavelengths higher than 200 nm; hence, it represents a cheap and readily available reference gas to set up Spectra for the in-field analysis. Nevertheless, the use of a non-standard gas as a reference raised the need for stringent automatic data analysis to readily identify potential air contaminations and exclude the resulting concentration measurements. The control of potential air contaminations is realized in two subsequent stages:

- Since the absorptions of UV-active compounds in the reference gas are subtracted to the absorption of the sample gas according to Equation 2, every spectrum is checked for the presence of negative absorption bands, especially in wavelengths range that does not show natural gas signals;
- After an automatic control that takes into consideration the threshold values and the expected pure components absorptions, spectra containing negative bands are automatically discharged.

The quantification of the various BTX compounds was optimized by varying the setup of the automatic acquisitions, changing their number and the acquisition time to minimize noise. Acquisitions were recorded as the average of 50 light readings, each with 0.1 s integration time. Direct absorptiometry of benzene, toluene, ortho-, meta-, and para-xylenes was performed by comparing the absorbance of the gas mixture at 200 nm with the absorption recorded with standard canisters at the same wavelengths. The LOD (level of detection) and the LOQ (level of quantification) have been calculated for benzene, toluene, and for the xylenes. The calculations were performed by obtaining the standard deviations of the absorbance values recorded for 45 blanks at the measurement wavelengths and setting 3 times this value as LOD and 10 times this value the LOQ in the selected range of analysis. According to these calculations and the experimental conditions selected for this work, Spectra gave the LOD and LOQ in mg over standard m${}^{3}$, as reported in Table 2.

### 2.2. Materials

All analytical gas samples for the in-house analysis of benzene, toluene, and the ortho-, meta-, and para-xylenes were obtained from Risam Gas with a concentration ranging from 10 to 52 mg/Sm${}^{3}$ in nitrogen. In-field gas samples were spilled directly from medium-pressure (3–5 bar) distribution pipelines with the consent of the distributors.

## 3. Results and Discussion

**Figure 2:**
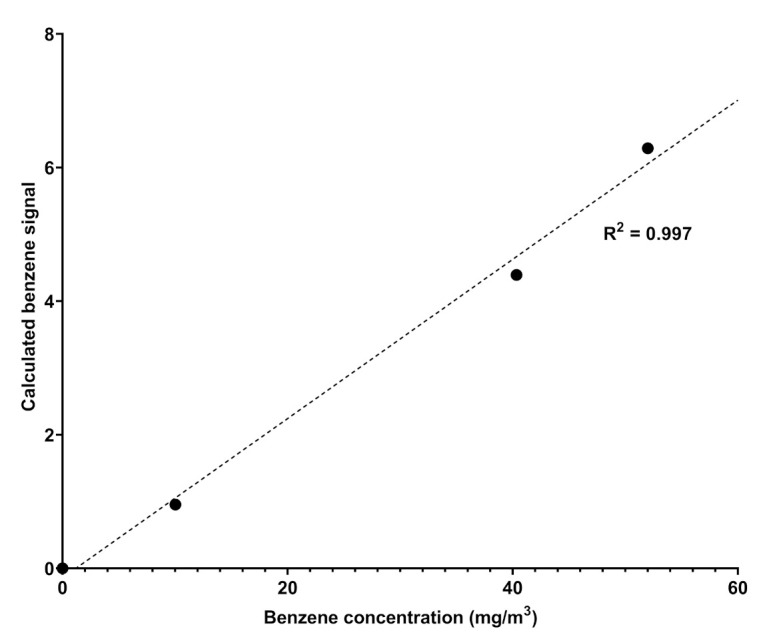
Calibration curve obtained by fitting the absorption values obtained by Spectra against the different concentration of benzene in nitrogen.

Spectra is a UV–visible spectrophotometric instrument able to detect some components of a natural gas mixture in the range 200–280 nm; the choice of this range of analysis rules out the absorptions of aliphatic hydrocarbons, which fall at higher energies. The instrument is positioned directly in the pipes and, through direct sampling, provides an analysis of the gaseous mixture over time, at established and regular intervals that allow constant remote monitoring of the content of some target components. In general, it provides UV–visible spectra in the established range of wavelengths whose absorbance profiles are compared with a database of spectra recorded for pure components (spectral modeling); the comparison allows the qualitative recognition of the components of the gaseous mixture and, ultimately, by taking the calibration line as reference (Figure 2), allows the quantitative measurements of selected components as, for example, sulfured odorants [18].

**Figure 3:**
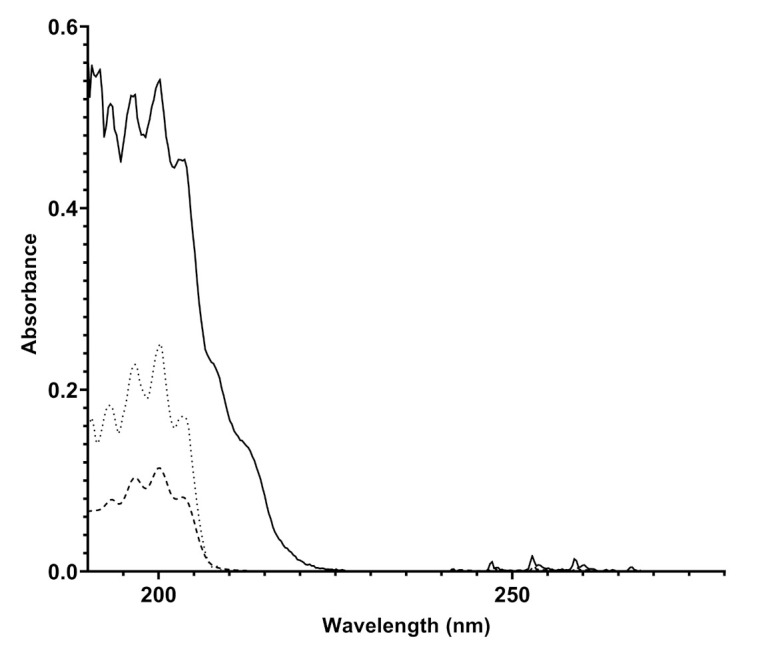
Overlapped UV spectra of a natural gas mixture detected by Spectra (bold line), of 10 mg/Sm${}^{3}$ benzene in nitrogen canister acquired by Spectra (dashed line), and an experimental UV spectrum taken from the literature (dotted line) [19].

The instrument is equipped with sophisticated electronic measurement, control, and communication systems in order to process and send the data to a platform that acts as a collector of both spectra and final measurement results (concentration in mg/Sm${}^{3}$ or ppm). The installation of Spectra instruments in different spots of a pipeline, but also in different natural gas distribution networks in and outside of Europe, allowed to observe differences in the composition of natural gas depending on the country of injection and the specific analyzed pipeline spot. The spectra produced in different pipelines have unequivocally highlighted the presence of benzene and benzenoids (BTX) in different concentrations, depending on the pipeline considered. An example of a spectrum produced by Spectra appears in Figure 3, compared both to the spectrum obtained with a canister of benzene in nitrogen 10 mg/Sm${}^{3}$ and with the spectrum of gaseous benzene reported in literature.

The attribution of the absorptions recorded by Spectra to benzene and benzenoids was carried out by analyzing the data reported in the literature. Benzene is a highly symmetric cyclic molecule consisting of six carbon atoms and six hydrogen atoms. The molecular structure is made of alternating double and single C-C covalent bonds, with the hydrogen atoms bonded to the carbon atoms by C-H σ-bonds. The absorptions of UV light are due to the π- or σ- molecular orbital electrons which are promoted to π* or σ* antibonding molecular orbitals. The presence of a different set of MO electrons is responsible for many transitions and, according to the symmetry rules, benzene, being a D_6h_ symmetry molecule, affords to two main sets of absorptions corresponding to ^1^A_1g_ → 1B2u and ^1^E_1g_ → 1B2u [20,21]. In particular, the detection of benzene is highlighted by the presence of finger-shaped absorption bands in the frequency range 230-270 nm due to valence shell ^1^A_1g_ → 1B2u electronic transitions; the absorption corresponds to about 4.7–4.9 eV energy [21] and another absorption at 160–190 nm due to the ^1^E_1g_ → 1B2u electronic transition. The overlapped spectra showed in Figure 3 display the same absorptions verifying the unequivocal presence of benzene in natural gas, likely with traces of other aromatic benzenoids. The quantitative determination of benzene in the pipeline was allowed by comparing the absorption spectra of the sample to known pure component spectra (spectral modeling) and then by referring to the calibration line recorded by Spectra with canisters of benzene with different concentrations in nitrogen, which is reported in Figure 2. The natural gas spectrum reported in Figure 3 was originated by a natural gas pipeline with a concentration of benzene corresponding to 9.83 ± 1.28 mg/m${}^{3}$.

Toluene and xylenes consist of benzene rings where one or two hydrogen atoms are substituted with one or two methyl groups, respectively. The presence of two methyl groups in the ring yields different isomers, according to numbering from one to six in the carbon atoms: 1,2-dimethyl-benzene is the ortho-xylene, 1,3-dimethyl-benzene is the meta-xylene, and 1,4-dimethylbenzene is the para-xylene. For the latter, the similitude of their molecular structures features this class of benzenoids as the most difficult to be discriminated. When methyl substituents appear in the aromatic ring, the absorptions are not drastically affected but some effects can be easily noticed; the absorption bands are broadened and red shifted as evidence that the symmetry of the electronic wavefunctions of the respective π MOs is not seriously affected upon methyl substitution. From theoretical calculations [22], the methyl substitution does not cause serious changes on the energy and relative order of the different occupied MOs, except for a lifting of degeneracy and a progressive decrease in the ionization energies due to the inductive effect of the methyls [23].

**Figure 4:**
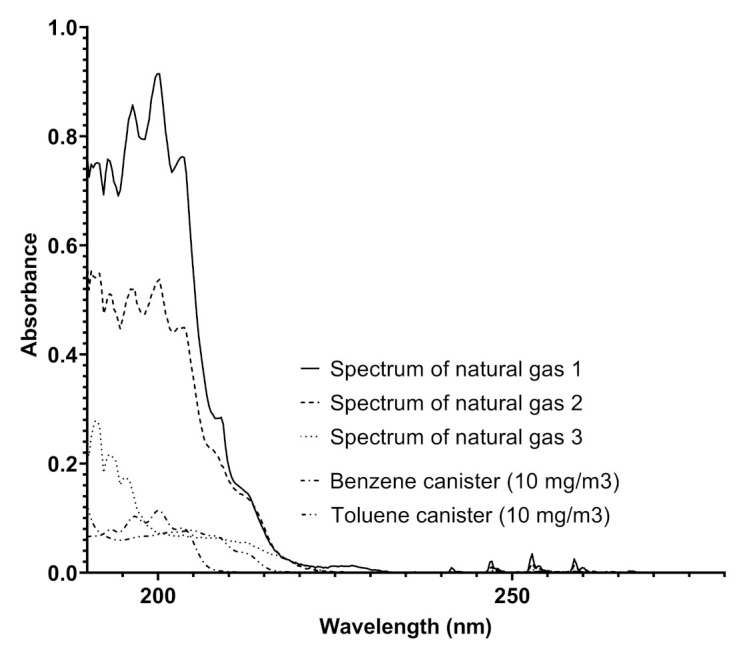
A comparison among the spectra obtained from a natural gas pipeline on different dates and the spectra of benzene and toluene recorded by Spectra using canisters of benzene and toluene (both 10 mg/Sm${}^{3}$) in nitrogen.

The UV–visible spectra produced by Spectra were analyzed by also considering the absorptions of benzenoids with an increasing number of methyl groups such as toluene and xylenes. A comparison of the spectra obtained in pipelines and benzene and toluene spectra is reported in Figure 4.

**Figure 5:**
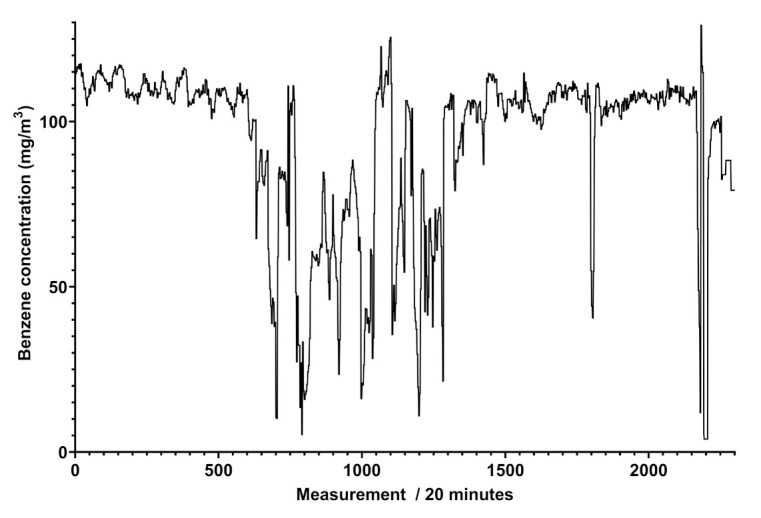
Graph of the benzene concentrations in mg/Sm${}^{3}$ obtained by Spectra (vertical axis) between 11 April and 13 May 2021 (horizontal axis), with a reading performed every 20 min in an extra EU natural gas pipeline. A total of 504 measurements correspond to an observation time of a week.

The analysis of the spectra reported in Figure 4 furnishes some details about the determination of different concentrations of benzene and toluene in the same pipeline, indicating the variability of the composition of the gaseous mixture with time, where the concentrations of the two aromatic compounds range from 0.31 to more than 80 mg/Sm${}^{3}$ in a single week. This analysis was highly relevant in another pipeline, situated in an extra European country, where the gas composition referred to these components was much more variable, and the trend of benzene concentration ranged up to 120 mg/Sm${}^{3}$. The trend of benzene concentration was monitored and the results over a month of measurements for a specific line is reported in Figure 5.

**Figure 6:**
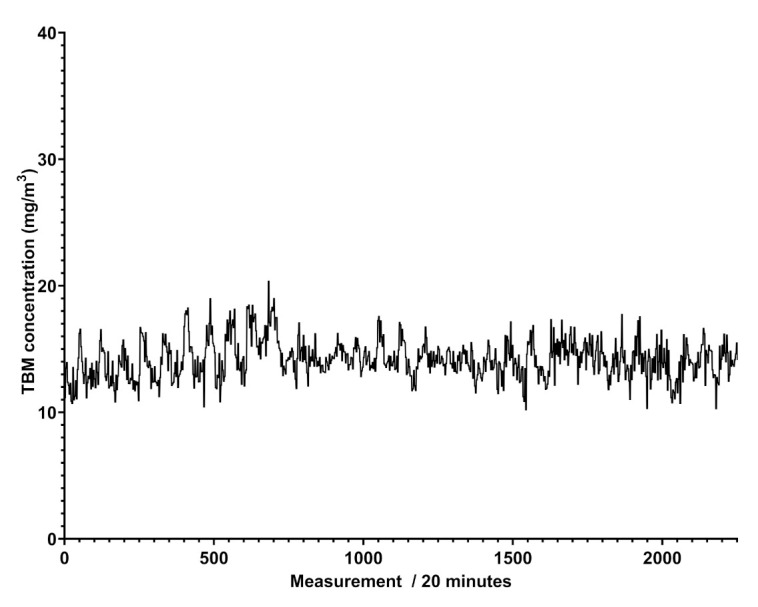
Plot of the determined odorant concentration over time in mg/m${}^{3}$.

Figure 5 shows that the trend of benzene’s concentration recorded in a natural gas pipeline is not regular and does not show cyclic events, different to what was attained for scheduled odorant injection in the pipeline, revealed by Spectra as peaks of concentration at given days and shown in Figure 6.

**Figure 7:**
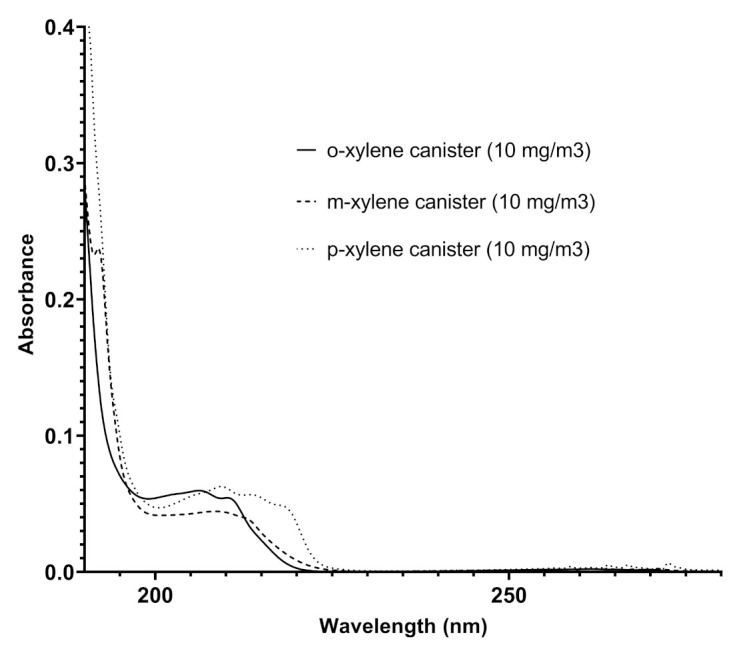
Overlapped spectra of ortho-xylene, meta-xylene, and para-xylene recorded by Spectra in canisters (the concentration of each xylene isomer was 10 mg/m${}^{3}$).

The analysis of the spectra recorded in different pipelines over time reveals additional functionalities for Spectra. In addition to benzene and toluene, other benzenoids were also detected: the ortho-, meta-, and para- xylenes. Their presence was ascertained following the redshift of the finger-shaped bands in the range 220–280 nm [24], appearing less defined in their vibronic structure, and by comparing the absorptions recorded by Spectra in the pipeline with those obtained by Spectra with canisters of different xylenes in nitrogen at established concentrations and shown in Figure 7. Some representative results in terms of contents of the different benzenoids are reported in Table 3.

The measurements reported in Table 3 are randomly taken in different natural gas pipelines to underline the different benzene levels in the same net of distribution. By observing the values of the concentrations of benzene and benzenoids in mg/Sm${}^{3}$ recorded in different pipelines (two belonging to European gas distributors and one from an extra Europe pipeline), it is possible to perceive the large variability of benzene and benzenoids concentrations in the same pipeline at different times (entries 1–5 of Table 2) as well as the different composition of the natural gas due to diverse provenience of the gas suppliers.

## 4. Conclusions

It is known that methane gas for civil purposes, either natural or originated by other processes, may contain aromatic compounds. The presence of benzene and similar compounds cannot be avoided but it might be limited by removing them when the concentration exceeds the limits consented by law. On the other hand, the determination of benzene, toluene, and xylenes in a hydrocarbon gaseous mixture is restricted to a few cases and mostly by using chromatographic methods. In this work, the measurement of benzene and benzenoids’ concentrations in a continuous modality by means of an in-field apparatus is described. The instrument consists of a robust UV spectrophotometer already skilled to simultaneously assay sulfured odorants [18]. Preliminary tests exhibit benzene contents in the pipelines never exceeding the law limits (see Figure 5 in example), promoting this instrument as a control device for the monitoring of many kinds of gaseous mixtures.

## Figures and Tables

**Table 1:** Comparative analysis of a standard canister containing 52 mg/m${}^{3}$ of benzene in nitrogen by independent laboratories using different methods of analysis and by Spectra.

	Benzene (mg/m^3^)	Relative Error [%]	Method of Analysis
Lab A	46.7	−10.19	GC-MS after solvent extraction
Lab B	47.85	−7.98	Detector tube (colorimetric analysis)
Spectra 1	52.16	0.31	UV–vis absorption spectroscopy
Spectra 2	52.99	1.90	UV–vis absorption spectroscopy

**Table 2:** LOD and LOQ values calculated for benzene and BTX.

	Benzene	Toluene	o-Xylene	m-Xylene	p-Xylene
LOD (mg/Sm${}^{3}$)	0.55	0.81	1.05	1.41	1.00
LOQ (mg/Sm${}^{3}$)	1.84	2.70	3.51	4.71	3.34

**Table 3:** Concentrations of benzene, toluene, and total xylenes in mg/Sm${}^{3}$ in different pipelines at given dates (EU1 and EU2 pipelines belong to European countries, Extra EU pipeline does not belong to European countries).

Pipeline Entries	Date (dd/mm/yyyy)	Benzene	Toluene	Total Xylenes	Notes
1	12/04/2019	6.03 ± 0.25	5.08 ± 0.42	1.10 ± 0.8	EU-1
2	14/04/2019	51.40 ± 0.18	24.32 ± 0.18	2.09 ± 1.75	EU-1
3	15/04/2019	25.42 ± 0.28	18.49 ± 0.66	3.84 ± 2.15	EU-1
4	23/06/2019	77.78 ± 0.73	28.25 ± 0.21	1.31 ± 0.03	EU-1
5	09/01/2020	7.41 ± 0.10	5.04 ± 0.22	16.84 ± 1.49	EU-1
6	27/02/2021	104.10 ± 2.64	52.32 ± 1.32	1.80 ± 0.23	Extra EU
7	11/03/2021	78.65 ± 3.78	42.05 ± 1.01	2.12 ± 1.67	Extra EU
8	15/03/2021	29.16 ± 0.23	14.20 ± 0.40	2.33 ± 0.84	Extra EU
9	25/03/2021	11.94 ± 0.28	6.64 ± 0.32	1.09 ± 0.70	Extra EU
10	12/05/2021	0.00	0.78 ± 0.57	24.90 ± 4.14	EU-2
11	12/05/2021	0.00	0.00	33.34 ± 6.09	EU-2
